# The Care of Cripples

**Published:** 1920-11-27

**Authors:** 


THE PUBLIC WELL-BEING—(*continued*).

### The Care of Cripples.

### PROGRESS IN TREATMENT AND EDUCATION.

The joint conference of the Invalid Children's Aid Association and the Central Committee for the Care of Cripples, to which I referred last week (writes a correspondent), did good service in calling public attention to the need for further organisation in the care of cripples and for greater financial help from the State, as well as by individual effort. I understand that the Government are to be directly approached on this subject, and particularly with a view to giving effect to the resolution passed by the conference that "the age at which grants are available for the treatment of cripples should be extended to the maximum age for continuous classes under the Education Act of 1918."

This resolution is the more likely to receive practical consideration at the hands of the Government, in view of the presence at the conference of Mr. Herbert Lewis, the Parliamentary Secretary of the Board of Education, who in his address showed that he is really in sympathy with the cause of crippled children. He himself declared that with proper treatment, combined with suitable education, 75 per cent. of the child cripples of the country could be turned into satisfactory workers. Expenditure of money on their treatment is not merely an act of merit, but a sound investment, because it means that thousands of children who would otherwise grow up to be a burden on their relatives and on the community will become useful and self-supporting citizens.

There is rather a tendency in some quarters to argue in these days that it is a sort of mistaken kindness to carry out extensive measures for the care of cripples, of insane and mentally defective persons, old people, or those stricken with incurable disease. They are a burden to the community, so the theory is, and to prolong their lives deliberately is to add to the burden and probably to create more disease, mental and physical, in the future. That is a limited and an inaccurate, as well as an inhumane, view. Apart from the importance of segregation in a large class of such cases, and the value to the race at large of having them under close scientific observation, it is obvious that if once we allow ourselves to cheapen our regard even for the most worthless or the most useless of human lives, we shall insensibly grow to cheapen our regard for human life in general, and so cease to occupy ourselves about preserving and strengthening it.

That is the reply I always make to the point of view of downright persons who say "The greatest kindness one can do for that wretched creature is to put him in a lethal chamber." At least such people should be ready to agree that it "pays" to turn a helpless cripple into a happy and self-supporting citizen. Great progress has been made on the right lines in the last twenty years, though a great work remains to be done. Since 1900 there have been established 123 schools, accommodating 9,000 children, and twelve residential schools, accommodating 540 children. Among them are a certain number of schools certified by a Board of Education. The Invalid Children's Aid Association has twenty-three branches in London, with thirty-seven affiliated country branches; eight homes are maintained by the Association, and there are over 33,000 children on the London books. The last report of the Chief Medical Officer of the Ministry of Health showed that there are 3,359 health visitors throughout the country and 1,754 maternity and infant welfare centres, of which 1,062 are municipal and 693 voluntary centres. It is in these centres that, more than has yet been realised, the real work of *prevention* may be looked for.

"Of all the pathetic spectacles which the world can furnish, that of suffering children is perhaps the most pathetic." All who have given their leisure or their lives to the work of caring for crippled children will endorse from the depths of their hearts this remark, in which Mr. Lewis summed up his attitude towards the cause which he appeared to champion with so much sincerity.

